# Dataset for analysis of computational fluid dynamics results from an industrial centrifugal fan

**DOI:** 10.1016/j.dib.2025.112269

**Published:** 2025-11-12

**Authors:** Matheus Costa Pereira, Mirelli de Castro Cesário, Anderson Paulo de Paiva

**Affiliations:** Federal University of Itajubá (UNIFEI). Avenida BPS, 1303, Pinheirinho, Itajubá/MG, 37500-903, Brazil

**Keywords:** Turbomachinery, Design of experiments, Response surface methodology, Numerical simulation

## Abstract

This dataset comprises 504 observations obtained from numerical simulation using Computational Fluid Dynamics for a real industrial application involving a turbomachine, specifically a centrifugal fan responsible for air recirculation in ovens operating at high angular velocity and temperature. Prior to geometry construction, a Design of Experiments strategy was employed, based on a Central Composite Design to generate a response surface. Four geometric input variables related to the centrifugal fan blades were considered: number of blades, blade entry angle, blade opening angle, and blade length. The design comprised 24 simulation trials, which, combined with 20 replicates generated by small mesh variations, resulted in 504 numerical simulations.

The entire process was executed in Ansys®, with Workbench used to organize and manage the project workflow from pre-processing to post-processing. Geometry modeling was carried out in SpaceClaim, while mesh generation was performed in Fluent Meshing using polyhedral elements, local refinements for blade throat regions and high-gradient zones, and quality control ensuring orthogonal quality above 0.150 and skewness below 0.950. Simulations were conducted under steady-state conditions with a Multiple Reference Frame at 5600 revolutions per minute, employing the k–ω Shear Stress Transport turbulence model and a Coupled solver scheme. Final analyses were conducted in Fluent and CFD-Post.

The simulations produced 35 output variables, encompassing static, dynamic, and total pressures; velocity magnitude and its axial, radial, and tangential components; turbulence quantities; turbulent Reynolds number; Large Eddy Simulation resolution estimate; torque; mass flow rate; blade mass; material cost; and a dimensionless performance index. For certain variables, minimum, mean, and maximum values are reported. The structured workflow and dataset documentation promote reuse and reproducibility. This dataset is suitable for statistical analysis, response surface modeling and simulation, sensitivity studies, comparison of turbulence models and mesh configurations, design optimization, data science applications, and replication of the Design of Experiments methodology in other Computational Fluid Dynamics contexts.

Specifications Table

**Table:** 

Subject	Engineering & Materials science
Specific subject area	Heat transfer in turbomachinery using Computational Fluid Dynamics.
Type of data	Table
Data collection	The data were generated using Ansys® software. Fan geometries were modeled in SpaceClaim, where the scenarios were defined through a Design of Experiments approach to vary the input variables related to the centrifugal fan blade configuration. CFD meshes were generated in the Fluent Meshing environment, applying orthogonal quality and skewness constraints to ensure high-quality grids. The numerical simulations were performed in Fluent, while post-processing was carried out in both Fluent and CFD-Post. Flow and performance characteristics such as mass flow rate, torque, efficiency, velocities, pressures, and turbulence quantities were extracted from the simulations.
Data source location	• Institution: Federal University of Itajubá; • City/Town/Region: Itajubá, Minas Gerais; • Country: Brazil.
Data accessibility	Repository name: GitHub – Centrifugal Fan Data [1] Data identification number: https://doi.org/10.5281/zenodo.17438392 Direct URL to data: https://github.com/Matheuscp98/CentrifugalFanData
Related research article	None

## 1 Value of the Data

• Systematic experimental design: Provides a systematically designed dataset based on a Central Composite Design (CCD), enabling efficient exploration of the design space of interest and identification of variable effects and interactions.;
• Wide range of responses: The dataset covers variables related to flow behavior as well as thermal performance indicators, enabling comprehensive analyses, scenario comparisons, and informed decision-making in contexts with conflicting objectives;
• High-fidelity Computational Fluid Dynamics (CFD): Each data point was generated through detailed numerical simulation, ensuring consistency and enabling replication or comparison with new simulations and confirmatory experiments;
• Data science applicability: The consistent structure supports predictive modeling, feature selection, dimensionality reduction, pattern recognition, and model validation;
• Transparent and replicable benchmark: From DOE planning to post-processing, the documented workflow makes the dataset comparable to new cases with different mesh configurations, turbulence models, and numerical settings, while supporting sensitivity studies;
• Industrial and academic relevance: Originating from a real-world problem, it provides a systematic framework to investigate how geometric changes affect flow behaviour and power requirements, fostering innovation and design optimization with the minimum necessary number of simulations;
• Assessment of CFD limitations: For the construction of the dataset, during the computational simulation stage, mesh independence tests, variations in boundary conditions, and the use of the k–ω SST turbulence model, widely applied in turbomachinery, were carried out. These elements allow the evaluation of the sensitivity of simulations to critical factors such as discretization and near-wall flow modeling, providing support for the verification and validation of numerical codes applied in turbomachinery;
• Comparability for similar studies: The detailed description of boundary conditions, input numerical parameters, and mesh quality criteria makes the dataset comparable to other simulations and future experiments. Thus, it can be used as a reference in intercomparison validation studies, enabling the analysis of the impact of different turbulence models, mesh variations, and numerical coupling strategies;
• Connection with other projects: The output variables, such as mass flow rate, torque, total pressure, and maximum velocity, can be compared with results from other turbomachinery projects reported in the literature, both from CFD simulations and experimental measurements. This enables the calibration of empirical models, the assessment of their applicability under different geometric conditions, and the proposal of adjustments when discrepancies are observed.

## 2 Background

Design of Experiments (DOE) provides a structured approach for planning and analysing experiments, extracting maximum information with fewer runs [2]. It also allows the simultaneous evaluation of multiple factors and their interactions [3]. Within this framework, Response Surface Methodology (RSM) is often applied to model and second-order nonlinear responses in complex engineering problems. One of these applications is Computational Fluid Dynamics (CFD).

CFD enables the numerical study of fluid flow and its interaction with solid boundaries, offering insights into flow patterns, pressure distribution, and thermal effects. Despite its relevance, CFD involves high computational cost and significant hardware requirements [[4], [5], [6]]. Simulations may also require long execution times [7,8], accurate boundary conditions aligned with experimental data [[9], [10]], and careful model selection to represent physical phenomena faithfully [11].

When combined, CFD and DOE improve the efficiency of geometric modelling, provide robust analyses, and reduce computational effort. Several studies have successfully applied a dual approach combining CFD and RSM a combined CFD–RSM framework [[12], [13], [14], [15], [16], [17]]. Within this framework, the dataset compiles tabular results from CFD simulations of a centrifugal fan used in an industrial context, characterized as an aerothermal turbomachinery problem. The methodological approach includes parametric model construction and systematic variation of blade geometric parameters through a response-surface CCD, allowing a structured investigation of input factors on flow response variables.

The resulting dataset supports a broad range of applications, including modeling studies, CFD methodology comparisons, Machine Learning (ML) tasks, statistical analyses, model verification, single- and multi-objective optimization, sensitivity studies, and academic training.

## 3 Data Description

The data presented in this article originate from a case study of a centrifugal fan applied in industrial ovens. The dataset includes four input variables (factors) and 35 output variables (responses), yielding a total of 504 observations obtained through CFD simulations. All simulations were carried out in Ansys Fluent® under steady-state conditions, adopting the k–ω SST turbulence model. This model is widely applied in turbomachinery because of its ability to capture flow separation and steep gradients in near-wall regions. Boundary conditions included pressure inlet and pressure outlet, both set with a turbulence intensity of 5 % and a turbulence viscosity ratio of 10. The rotor rotation was imposed at 5600 rpm around the z-axis using the Multiple Reference Frame (MRF) approach, while the solid walls were treated as adiabatic with no-slip conditions.

The responses are provided in different formats: some correspond to raw values directly extracted from the simulations, while others are reported as summary statistics, such as minimum, mean, and maximum values, calculated within the fluid domain. The variables cover mass, mass flow rate, torque, performance, cost, pressures, velocity components, and turbulence quantities. A detailed description of the input and output variables included in the centrifugal fan dataset is provided in Table 1.

**Table 1:** Description of input and output variables for the centrifugal fan study.

Variable	Type	Abbreviation	Unit	Description
Number of Blades	Input	NB	adm	Number of impeller blades.
Blade Entry Angle	Input	BEA		Blade entry angle, measured in the blade plane relative to the local tangential direction at the inlet radius.
Blade Opening Angle	Input	BOA		Geometric angle opening or lean of the impeller blade, in the blade plane, referenced to the local radius (meridional plane).
Blade Length	Input	BL	mm	Blade length along the radius.
Blade Mass	Output	BM	kg	Unit mass of the blade.
Mass Flow Rate	Output	MFR	kg/s	Mass flow rate of the fluid passing through the cross-sectional area per unit time; in this case, the air moved by the fan.
Torque	Output	T	N·m	Amount of torque required to rotate the fan blades.
Performance Index	Output	PI	adm	Dimensionless indicator that evaluates the overall performance of the fan, combining variables such as mass, flow rate, and torque.
Material Cost	Output	MC	USD	Cost associated with the material of the rotating assembly for fan construction; manufacturing costs not included.
Static Pressure Minimum	Output	SP_min	Pa	Minimum static pressure in the fluid domain/region of interest.
Static Pressure Mean	Output	SP_mean	Pa	Mean static pressure in the fluid domain/region of interest.
Static Pressure Maximum	Output	SP_max	Pa	Maximum static pressure in the fluid domain/region of interest.
Dynamic Pressure Minimum	Output	DP_min	Pa	Minimum dynamic pressure in the fluid domain/region of interest.
Dynamic Pressure Mean	Output	DP_mean	Pa	Mean dynamic pressure in the fluid domain/region of interest.
Dynamic Pressure Maximum	Output	DP_max	Pa	Maximum dynamic pressure in the fluid domain/region of interest.
Total Pressure Minimum	Output	TP_min	Pa	Minimum total pressure in the fluid domain/region of interest.
Total Pressure Mean	Output	TP_mean	Pa	Mean total pressure in the fluid domain/region of interest.
Total Pressure Maximum	Output	TP_max	Pa	Maximum total pressure in the fluid domain/region of interest.
Velocity Magnitude Mean	Output	VMAG_mean	m/s	Mean velocity magnitude in the domain/region of interest.
Velocity Magnitude Maximum	Output	VMAG_max	m/s	Maximum velocity magnitude in the domain/region of interest.
**Variable**	**Type**	**Abbreviation**	**Unit**	**Description**
Axial Velocity Minimum	Output	AV_min	m/s	Minimum axial velocity component (along the rotation axis).
Axial Velocity Mean	Output	AV_mean	m/s	Mean axial velocity component (along the rotation axis).
Axial Velocity Maximum	Output	AV_max	m/s	Maximum axial velocity component (along the rotation axis).
Radial Velocity Minimum	Output	RV_min	m/s	Minimum radial velocity component (from the axis outward).
Radial Velocity Mean	Output	RV_mean	m/s	Mean radial velocity component (from the axis outward).
Radial Velocity Maximum	Output	RV_max	m/s	Maximum radial velocity component (from the axis outward).
Tangential Velocity Minimum	Output	TV_min	m/s	Minimum tangential/swirl velocity component.
Tangential Velocity Mean	Output	TV_mean	m/s	Mean tangential/swirl velocity component.
Tangential Velocity Maximum	Output	TV_max	m/s	Maximum tangential/swirl velocity component.
Turbulent Kinetic Energy Minimum	Output	k_min	m²/s²	Minimum turbulent kinetic energy (k) in the domain.
Turbulent Dissipation Rate ε Minimum	Output	eps_min	m²/s³	Minimum turbulent dissipation rate (ε) in the domain.
Turbulent Dissipation Rate ε Mean	Output	eps_mean	m²/s³	Mean turbulent dissipation rate (ε) in the domain.
Turbulent Dissipation Rate ε Maximum	Output	eps_max	m²/s³	Maximum turbulent dissipation rate (ε) in the domain.
Turbulent Dissipation Rate ω Minimum	Output	omega_min	1/s	Minimum specific dissipation rate (ω) in the domain.
Turbulent Dissipation Rate ω Maximum	Output	omega_max	1/s	Maximum specific dissipation rate (ω) in the domain.
Turbulent Reynolds Number Minimum	Output	*Re*_min	adm	Minimum turbulent Reynolds number in the domain.
Turbulent Reynolds Number Mean	Output	*Re*_mean	adm	Mean turbulent Reynolds number in the domain.
Large Eddy Simulation Resolution Estimate Mean	Output	LES_mean	adm	Mean value of the Large Eddy Simulation (LES) resolution indicator.
Large Eddy Simulation Resolution Estimate Maximum	Output	LES_max	adm	Maximum value of the Large Eddy Simulation (LES) resolution indicator.

The minimum, mean, and maximum values reported for pressures, velocities, and turbulence quantities were extracted as volumetric statistics within the fluid domain. Mass flow rate was computed at the outlet surface, while torque was obtained from the integration of forces acting on the rotor blades. The velocity components (axial, radial, and tangential) were defined relative to the fan’s axis of rotation. The turbulent Reynolds number and the LES resolution indicator were calculated using the standard formulations available in Fluent, ensuring consistency and reproducibility across all simulations.

Convergence of each simulation was ensured by monitoring the stabilization of integral quantities, including torque, mass flow rate, and total pressure variation, with a tolerance of 1 % over a sliding window and a mass imbalance below 0.2 % between inlet and outlet. A mesh independence study was conducted using three refinement levels, showing differences below 2 % between coarse and refined grids for the reference case. All final meshes satisfied the recommended quality thresholds, with skewness lower than 0.95 and orthogonal quality greater than 0.15.

The dataset and supplementary files are publicly available in a GitHub repository [1]. The repository is structured to support data usability, provide clear descriptions of the variables, and facilitate their application in ML, statistical analyses, physical model development and validation, performance optimization, sensitivity studies, and academic training.

Regarding its organization, the repository includes both general usage guidelines and direct access to the dataset. In addition to the processed data, it contains the experimental design adopted to generate the CFD scenarios, ensuring transparency and reproducibility

Within the repository, the following files are provided:

• README.md: Describes the purpose of the dataset, how it was generated, the files included, and usage instructions;
• fan_doe.csv: Contains the CCD-type experimental design with variation levels for the four input variables, presented in both coded and uncoded formats;
• fan_dataset.csv: Main file containing 504 rows, 4 columns corresponding to input variables, and 35 columns corresponding to output variables;
• fan_abbreviation.csv: Maps abbreviations to the full variable names and their respective units, expressed in the International System of Units (SI).

## 4 Experimental Design, Materials and Methods

**Fig. 1:**
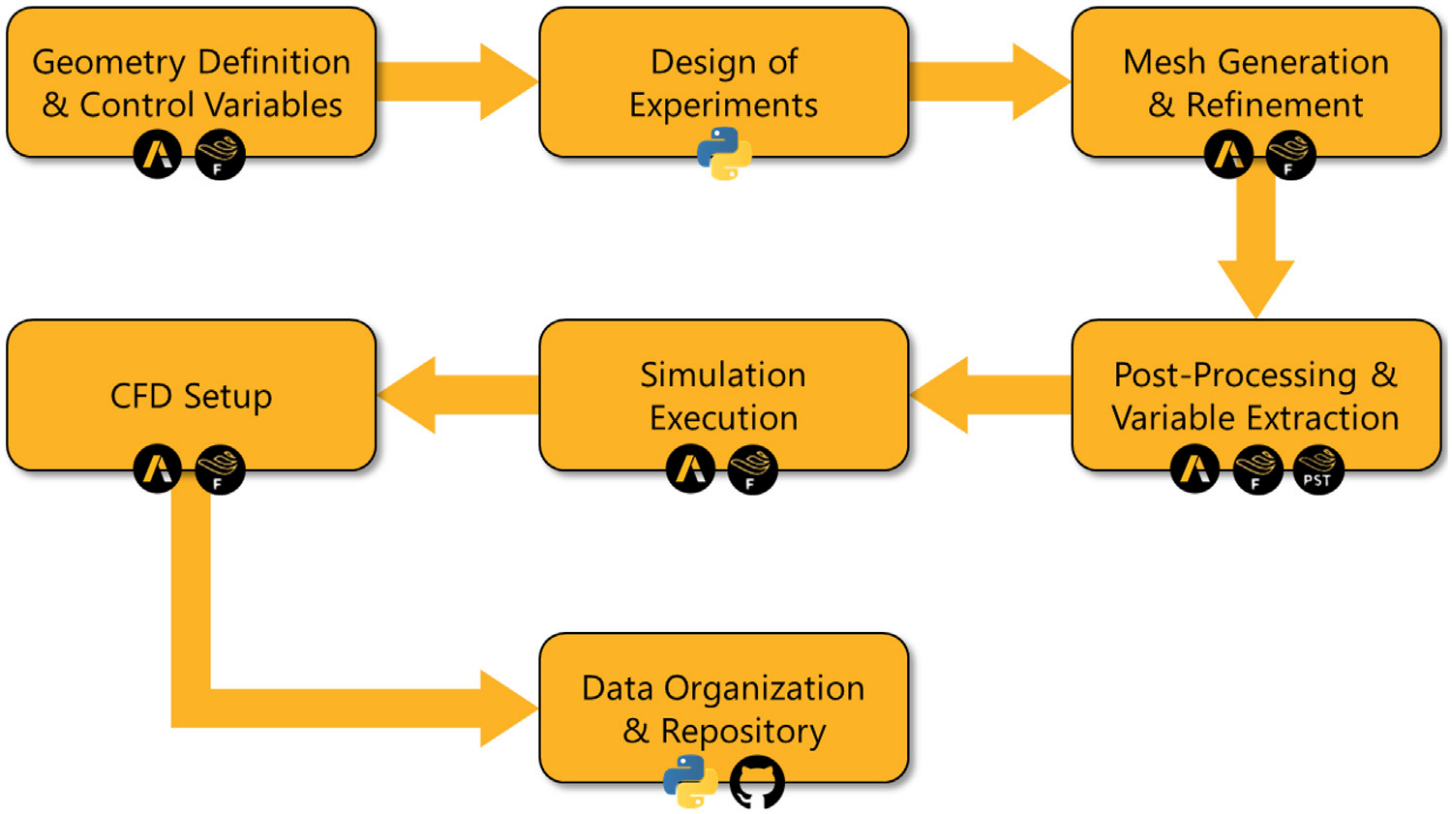
Methodological framework for dataset construction.

*Source:* Prepared by the author (2025).

To facilitate the understanding of the dataset construction, Fig. 1 presents a framework with the main stages of the process, ranging from geometry modeling to the post-processing of results. Each stage is associated with the corresponding Ansys® module or with the use of the Python programming language.

### 4.1 Experimental design and materials

The dataset was obtained through CFD simulations in Ansys®, applied to a centrifugal fan designed for air recirculation in industrial ovens, operating under high temperatures and high angular velocity. The rotor of the centrifugal fan, manufactured from austenitic stainless steel AISI 304 (American Iron and Steel Institute), operates at 5600 rpm (revolutions per minute). This material was selected for its high corrosion resistance, good formability, weldability, and thermal stability in harsh environments.

For all projects, with a blade thickness of 0.800 mm (millimeters), a blade width of 44.600 mm, and outer diameter of the volute cylinder of 198.000 mm were kept constant. In addition to these fixed parameters, controllable geometric variables were explored through experimental design:

• Number of blades (NB – x_1_): integer variable;
• Blade entry angle (BEA – x_2_): blade entry angle;
• Blade opening angle (BOA – x_3_): blade opening or geometric inclination angle;
• Blade length (BL – x_4_): radial blade length.

**Fig. 2:**
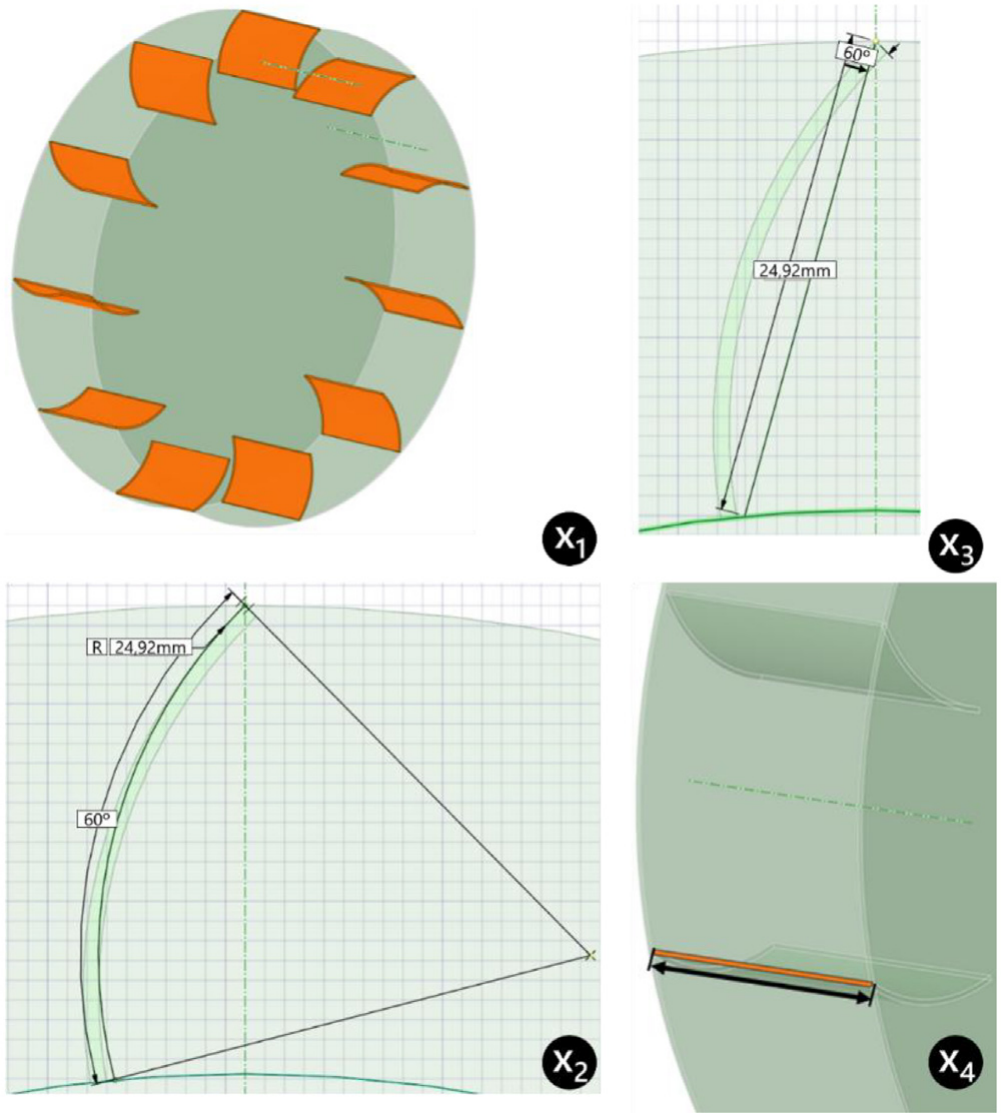
Measurement procedure of control variables.

*Source:* Prepared by the author (2025).

**Fig. 3:**
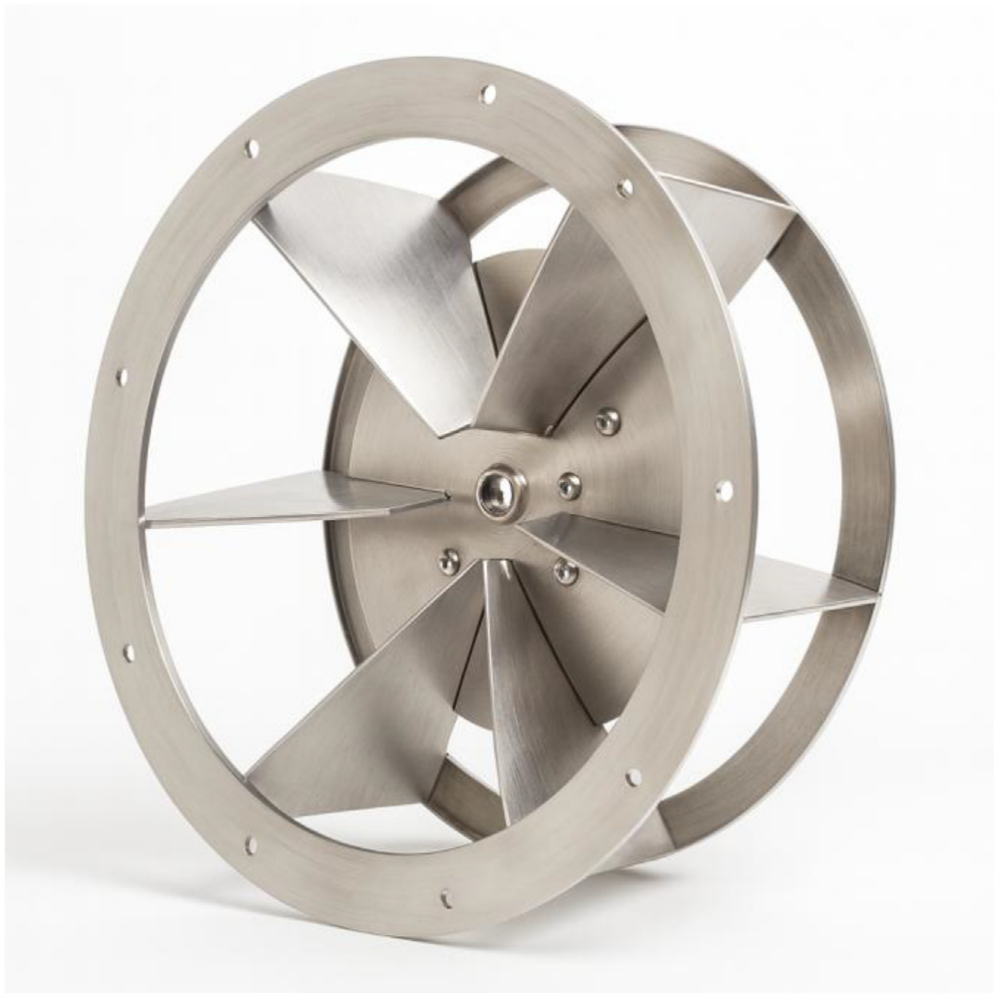
Reference fan for the study.

*Source:* Prepared by the author (2025).

**Fig. 4:**
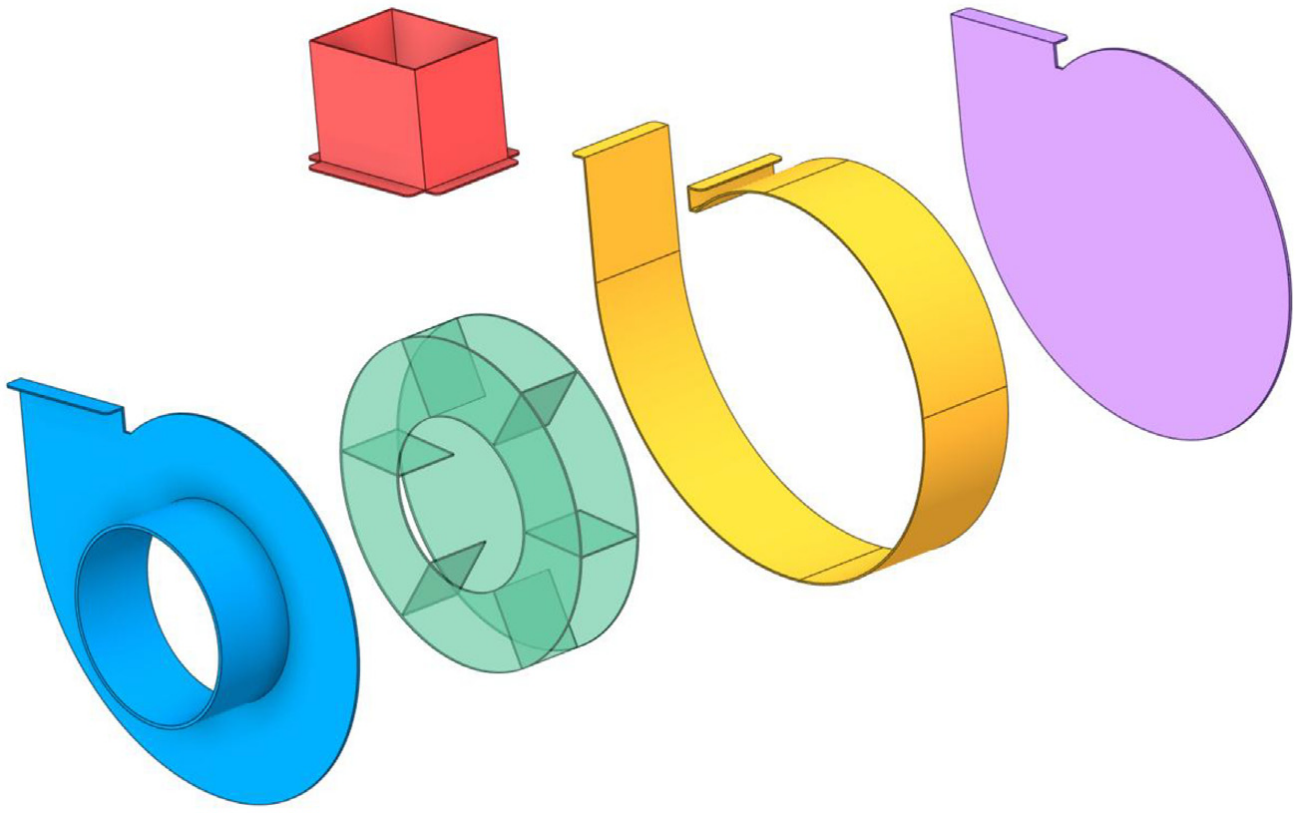
Exploded view of the reference fan assembly.

*Source:* Prepared by the author (2025).

Fig. 2 shows the procedure for measuring the control variables in SpaceClaim, illustrating how each parameter is defined within the Computer-Aided Design (CAD) environment. The reference fan, used as the baseline case and for internal validation, consists of six straight blades without inclination, as shown in Fig. 3. Fig. 4 provides an exploded view of the fan assembly.

The experimental design followed a CCD within a DOE, suitable for fitting response surfaces with linear, interaction, and quadratic terms using a reduced number of runs. Four continuous factors (NB, BEA, BOA, and BL) were considered, comprising 16 factorial points, 7 axial points, and 1 central point, totaling 24 runs with a rotational α of 2. One theoretical axial point implied a geometry with only two blades, which violated industrial design constraints, and was therefore removed.

To improve statistical robustness and evaluate the numerical sensitivity of the numerical model parameters the simulation parameters, 20 replicates were performed, introducing small controlled mesh variations, resulting in 504 simulations. The response variables included cost, performance, mass, mass flow rate, torque, velocity and pressure fields, and turbulence quantities.

### 4.2 Simulation workflow

**Fig. 5:**
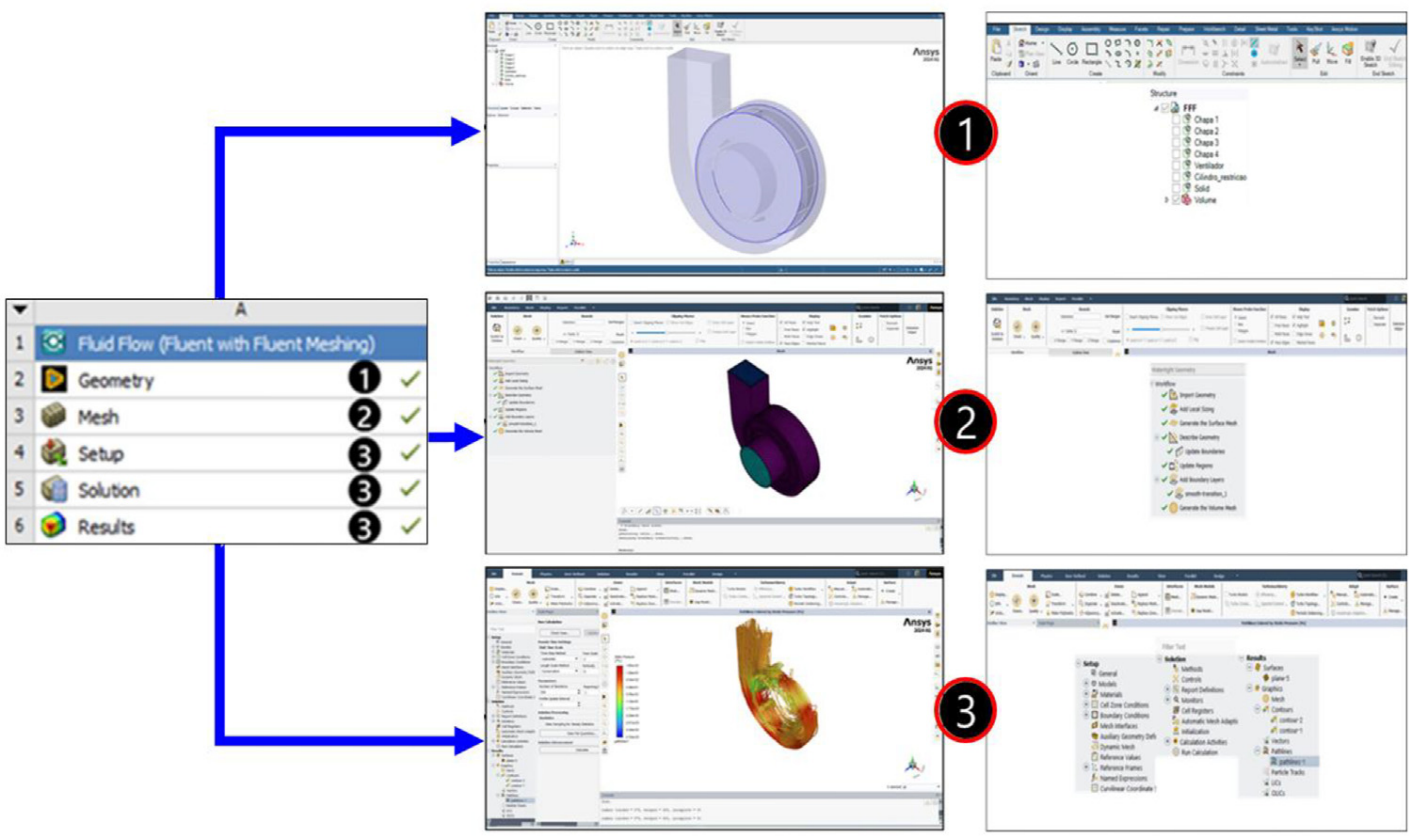
Stages of the numerical study in Ansys®: geometry, mesh, setup, solution, and results.

*Source:* Prepared by the author (2025).

The entire simulation workflow was carried out entirely within Ansys®, encompassing the configuration of the Workbench for project management, parametric modelling in SpaceClaim, mesh generation and refinement in Fluent Meshing, execution of simulations in Fluent, and, finally, analysis and extraction of results in both Fluent and CFD-Post. The complete simulation workflow in Ansys®, from parametric modelling to post-processing, is shown in Fig. 5.

### 4.3 Parametric modeling

Parametric modeling was performed in SpaceClaim with direct adjustments to NB, BEA, BOA, and BL. While Discovery or other CAD software could have been used, SpaceClaim was selected due to its native integration with the simulation environment.

### 4.4 Meshing

Meshing was performed in Fluent Meshing, using volumetric filling with polyhedral elements after cut-cell/hexcore generation, which improves average quality and gradient smoothness without excessively increasing the element count.

• Global sizing: Base size with automatic local refinements using Size Functions of Curvature (to reduce element size in regions with small curvature radius) and Proximity (to adapt element size to the minimum gap between adjacent surfaces). The scope was set to Proximity to Edges, prioritizing blade edges and narrow gaps.
• Boundary layers: Inflation layers with smooth transitions (gradual thickness progression), added in fluid regions adjacent to walls, growing only from selected walls. Target *y*+ was maintained between 1 and 5 for wall integration with the k-ω SST model.
• Quality metrics: Orthogonal quality greater than 0.150 and skewness <0.950, and no cells with quality lower than 0.05.

A mesh independence study was conducted for the baseline case, with three refinement levels (coarse, medium, fine) adjusting minimum and maximum size and growth rate. The absolute percentage differences between coarse and fine meshes for torque and total pressure variation were below 2 percent, justifying the choice of the “standard” resolution used for the 24 runs and replicas. Variations applied in replicas remained within this range, ensuring reproducibility.

### 4.5 Simulation setup

Simulations were performed in Fluent under steady-state conditions, using a pressure-based solver, pressure-velocity coupling via the Coupled scheme, and second-order discretizations for pressure, momentum, energy, and turbulence equations. The flow in the blade passage region was modeled with Multiple Reference Frame (MRF) at 5600 rpm around the z-axis. The k-ω SST (Shear Stress Transport) turbulence model was adopted due to its ability to accurately represent boundary layer transitions and flow separations under high gradients near walls.

The boundary conditions were as follows: Inlet using pressure inlet with normal direction to the surface, turbulence intensity of 5 percent, and turbulence viscosity ratio of 10; Outlet using pressure outlet with reference gauge pressure and the same turbulence viscosity ratio of 10 for backflows; and Walls with no-slip condition, adiabatic behavior, and no explicit roughness.

The Warped-Face Gradient Correction option was enabled to reduce gradient reconstruction errors on non-planar faces. The explicit relaxation factors were 0.50 for pressure and momentum, 1.00 for density, body forces, and turbulence viscosity, and 0.75 for k and ω.

Convergence was monitored using residuals for continuity, velocity components (x, y, z), and the k and ω equations. In addition to residual criteria, integral monitors for mass flow rate, torque, and pressure variation were used, requiring relative stabilization within 1 percent over a sliding window and a mass imbalance lower than 0.2 % between inlet and outlet.

### 4.6 Results extraction and visualization

After convergence, results were extracted in Fluent and CFD-Post, including pressures (static, dynamic, and total), velocities (magnitude and axial, radial, and tangential components), turbulence quantities (k, ε, ω), turbulent Reynolds number, LES resolution estimate, torque, mass flow rate, mass, and performance index. For each variable, minimum, mean, and maximum values were recorded.

**Fig. 6:**
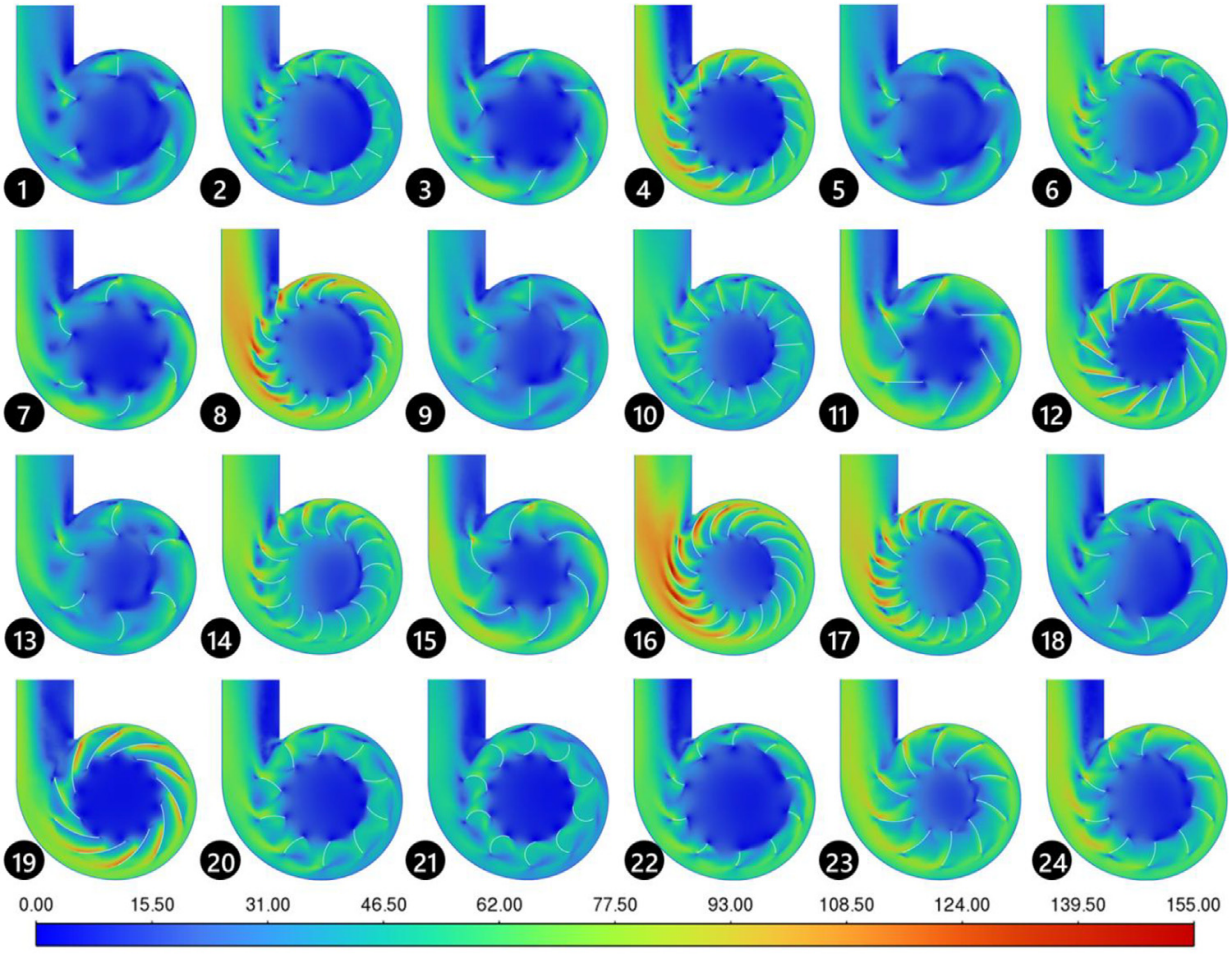
Velocity contour distribution on the central plane for 24 CCD experiment.

*Source:* Prepared by the author (2025).

Fig. 6 shows the contour lines corresponding to the mean velocity magnitude obtained from 24 numerical simulations of the CCD. For comparative analysis, a central plane was defined in the fan’s operating region, allowing observation of how velocity is distributed throughout the flow in each experiment. The same scale, ranging from 0 to 155 m/s, was applied to all simulations to facilitate the visualization of differences in the velocity field across the various scenarios.

Finally, in addition to Ansys®, the Python programming language was used to organize the DOE, automate the consolidation of the 504 simulations, and compile the final results tables. Process reproducibility was ensured through full parameterization in Ansys®, covering all stages from pre-processing to post-processing.

## Limitations

The limitations stem from the initial experimental design: it considers four geometric factors while keeping material, angular velocity and thermal conditions fixed at boundary conditions. Changes in parameter levels, operating and boundary conditions, the turbulence model or the application context can affect the responses and yield different outcomes. The released data consist of statistical summaries, such as minimum, mean, and maximum values, without spatial fields or full-time histories, which limits uses that require spatio-temporal resolution. Although a mesh-independence study was conducted, the public dataset does not include mesh variations or their results.

## Ethics Statement

If none of the above, please include a statement confirming that the authors have read and follow the ethical requirements for publication in Data in Brief and confirming that the current work does not involve human subjects, animal experiments, or any data collected from social media platforms.

## CRediT Author Statement

**Matheus Costa Pereira:** Conceptualization, Methodology, Data curation, Writing – original draft**; Mirelli de Castro Cesário:** Conceptualization, Writing – original draft; **Anderson Paulo de Paiva:** Supervision, Writing – review & editing**.**

## Data Availability

GitHubCentrifugalFanData (Original data).
